# Nanobody Technology for Mycotoxin Detection: Current Status and Prospects

**DOI:** 10.3390/toxins10050180

**Published:** 2018-04-29

**Authors:** Ting He, Jiang Zhu, Yao Nie, Rui Hu, Ting Wang, Peiwu Li, Qi Zhang, Yunhuang Yang

**Affiliations:** 1 State Key Laboratory of Magnetic Resonance and Atomic Molecular Physics, National Center for Magnetic Resonance in Wuhan, Wuhan Institute of Physics and Mathematics, Chinese Academy of Sciences, Wuhan 430071, China; ht210@wipm.ac.cn (T.H.); jiangzhu@wipm.ac.cn (J.Z.); nieyao@wipm.ac.cn (Y.N.); hurui@wipm.ac.cn (R.H.); 2 Oil Crops Research Institute of the Chinese Academy of Agricultural Sciences, Wuhan 430062, China; wangting963@163.com

**Keywords:** Nanobody, Mycotoxins, Immunoassay, Food safety

## Abstract

Mycotoxins, which are toxic, carcinogenic, and/or teratogenic, have posed a threat to food safety and public health. Sensitive and effective determination technologies for mycotoxin surveillance are required. Immunoassays have been regarded as useful supplements to chromatographic techniques. However, conventional antibodies involved in immunoassays are difficult to be expressed recombinantly and are susceptible to harsh environments. Nanobodies (or VHH antibodies) are antigen-binding sites of the heavy-chain antibodies produced from Camelidae. They are found to be expressed easily in prokaryotic or eukaryotic expression systems, more robust in extreme conditions, and facile to be used as surrogates for artificial antigens. These properties make them the promising and environmentally friendly immunoreagents in the next generation of immunoassays. This review briefly describes the latest developments in the area of nanobodies used in mycotoxin detection. Moreover, by integrating the introduction of the principle of nanobodies production and the critical assessment of their performance, this paper also proposes the prospect of nanobodies in the field of food safety in the foreseeable future.

## 1. Introduction

Mycotoxins are toxic metabolites produced by fungal species that have adverse effects on humans, animals, and crops, resulting in significant medical costs caused by mycotoxin-based diseases and large economic losses in international trade [1]. Aflatoxins (AFs), ochratoxins (OTs), zearelenone (ZEN), fumonisins (FBs), deoxynivalenol (DON), and other trichothecenes are the major mycotoxins of greatest agro-economic importance [2]. In light of the wide spread of mycotoxins, many countries set action regulations for mycotoxins in various food and foodstuff. Increasingly sensitive and effective determination technologies for mycotoxins surveillance are required. Table 1 displays some predominant mycotoxins and their major fungal producers, contaminated hosts, toxic effects [3], and their maximum permitted levels in foods according to European legislation.

There have been many efforts related to mycotoxin determination, typically using high-performance liquid chromatography (HPLC) [4], liquid chromatography-tandem mass spectrometry (LC-MS/MS) [5], and immunoassays [6,7]. Among these methods, immunoassays have been regarded as useful supplements to chromatographic techniques due to their merits in sensitivity, specificity, and low cost [8]. Large molecules can be easily determined by a sandwich-format immunoassay [9,10]. Small analytes, such as mycotoxins, are not large enough to be simultaneously recognized by two antibodies, so the competitive immunoassay formats are normally used, which need a hapten to compete with the target analyte for binding to the antibody [11].

**Figure 1:**
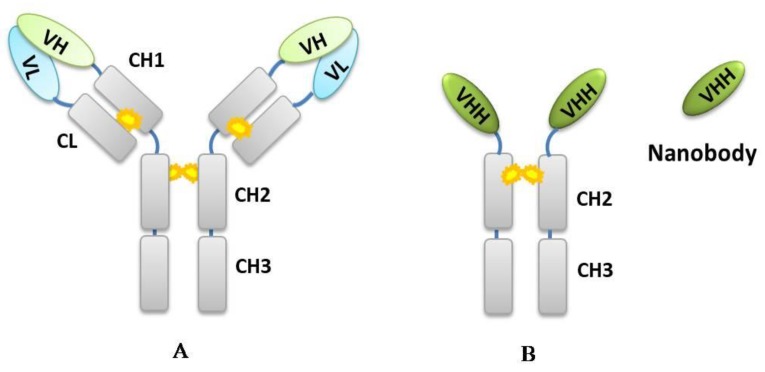
Schematic representation of a (A) conventional antibody (IgG) and (B) heavy chain camelid antibody. The isolated variable domain of the latter is called nanobody.

A large number of antibodies against mycotoxins have been developed, including polyclonal antibodies (pAbs) [15,16], monoclonal antibodies (mAbs) [17,18], and recombinant antibodies (rAbs) [19,20]. Since heavy-chain-only antibodies (HcAbs) that are naturally devoid of light chain and lacking the CH1 domains were first discovered in the serum of the camel (*Camelus dromedarius*) [21], camelid antibodies have gained increasing attention due to their convenience in biotechnological applications. While conventional mAbs and pAbs are composed of two heavy and two light chains with variable domains of heavy chain (VH) and light chain (VL) contributing to form a paratope of the antibody, the camelid HcAbs are composed of only heavy chains, of which the antigen-binding sites are formed only by the variable domain of the heavy chain (VHH) [22]. These VHHs can be expressed recombinantly and have been termed VHH antibodies, or single domain antibodies (sdAbs) [23]. Due to the nanometer-scale size (4 nm × 2.5 nm × 3 nm) of the VHH antibodies, they are also referred to as nanobodies (Nbs) by a biopharmaceutical company, Belgium Ablynx [24]. The representative structures of conventional antibody and nanobody derived from camelid HcAb are diagramed in Figure 1.

Sequence analysis and elucidation of the crystal structure have revealed several structural features of Nb fragments. The single domain nature of Nbs provides many advantages over conventional antibodies and other recombinant antibody fragments such as antigen-binding fragment (Fab), variable fragment (Fv), and single-chain variable fragment (scFv) [25,26,27]. The most characteristic feature of Nbs is the presence of amino acid substitutions in the framework-two region (FR2), making Nbs more soluble than conventional antibody fragments [28]. The extended flexible complementarity-determining region 3 (CDR3) makes Nbs capable of recognizing antigenic sites that are normally inaccessible or cryptic for pAbs and mAbs such as enzyme active sites and cryptic viral epitopes [29,30]. In particular, their small size (12~15 kDa), ease of cloning and expressing in various expression systems, high solubility and stability, resistance to proteolysis, and facile genetic manipulation, make Nbs an excellent alternative to producing immunoreagents for immunoassays [31]. For example, Nbs can be used in biosensors with higher immobilizing densities [32], used for affinity purification medication [33], used in the design of anti-idiotypic antibodies [34] and as modular building units for multivalent or multifunctional constructs [35]. 

Nanobody technology has been initially applied in diagnostic and therapeutic areas [36,37,38]. To date, most reported biotechnological applications of Nbs refer to proteins or other macromolecular antigens, while rare reports are related to Nbs used against small hapten (molecular weight below 1500 Da) for the reason of the sensitivities of most assays at the μM range that are somewhat unfavorable for practical application [39,40,41,42]. However, a growing number of studies have demonstrated that highly-sensitive Nbs maybe successfully isolated by sophisticated design of the selection procedure from the small subpopulation of high-affinity Nbs pool. There have been some successful examples regarding the use of Nbs to effectively recognize small molecules. Those compounds, such as the mycotoxins AFB_1_, OTA, and 15-acetyl-DON, and some environmental contaminants, were reviewed by Bever et al. [43].

Recently, there have been various forms of Nbs used in mycotoxin immunodetections. In addition to the hapten-specific Nbs commonly used as primary antibodies involved in immunoassay, anti-idiotypic Nbs (AI-Nbs), which are generated from mAbs-immunized camelid and which are capable of targeting the antigenic determinants of the primary antibodies, can be used for immunoassays as surrogates for competing antigens and can significantly improve the sensitivity of the assays [44]. For many analytical and diagnostic applications, Nbs have been directly used as phage-borne antibody fragments, relying on the multiple copies of M13 phage particles to increase the assay sensitivity [45]. Likewise, the phage DNA can be used for ultrasensitive detection of the phage-borne antibody by utilizing a combination of real-time polymerase chain reaction (RT-PCR) in different immunoassay formats, which widens the dynamic range of the assay by several orders of magnitude [46,47].

Considering the predominant traits of Nbs, they could meet the field-testing requirement of reagents with greatly enhanced stability for analytical methods and become promising alternative immunoreagents for mycotoxin detection. However, few reviews have provided detailed summaries of the development of the Nb-based immunoassays utilized for mycotoxin determination. This review focuses on the introduction of Nb technology for mycotoxin detection in the field of food safety and refers to recently reported studies, integrating the introduction of the principle of Nb development and providing a detailed description of their performance. This paper also proposes the prospect of Nbs in the area of analytical chemistry in the foreseeable future.

## 2. Principle of Developing Chemical-Specific Nb

Recombinant antibody techniques were used to create phage-displayed Nb libraries. To take advantage of the exquisite in vivo antibody-affinity maturation process, animals are often immunized prior to antibody isolation. It was noted that immune libraries lead more directly to anti-hapten Nbs with higher affinity and sensitivity than that of nonimmune libraries [43], although a few Nbs against small molecules (e.g., picloram [39] and auxin [40]) and AI-Nbs have been selected from native library [48,49,50].

Members of the Camelid family, including dromedary, llamas, and alpaca, are easily immunized following standard procedures [51]. Small haptens are compounds with low molecular weight and low immunogenic, customarily conjugated to a carrier protein (e.g., bovine serum albumin, BSA; ovalbumin, OVA; keyhole limpet hemocyanin, KLH, etc.) to induce an immune response. After immunization with weekly boosts, the total RNA is extracted from peripheral blood lymphocytes isolated from whole blood, and its cDNA synthesized using reverse transcriptase techniques [52]. The Nb-encoding fragments from IgG2 and IgG3-a subset of IgG antibodies lacking light chains are amplified and cloned into M13 phagemid vector to construct the phage-displayed Nb library. The resulting phage-Nb library can be used for panning target-specific binders.

**Figure 2:**
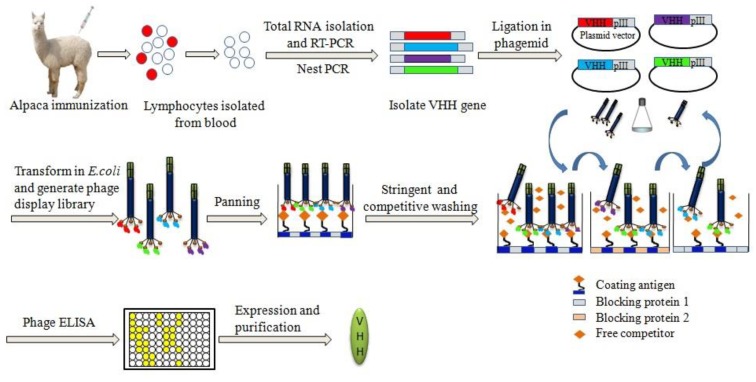
Schematic representation of the construction of phage-displayed nanobody library and the strategy of panning for the high-affinity Nbs isolation.

The panning strategy of improving selection pressure has been proved to be valid in isolating positive phage-Nb clones with favorable affinity and sensitivity [53,54,55]. Non-specific phage-Nbs were minimized by increasingly stringent washings and desorption of phage input/output to the carrier protein. Moreover, blocking reagents should be switched alternatively in each panning process which is beneficial to minimize the nonspecific absorption. There are two regular methods for the elution of phage binding to the immobilized target in the process of biopanning. One is polar elution (for example, 0.2 M glycine-HCl pH 2.2; or 0.1 M triethylamine pH 11.0 for elution of phage-Nb), which is frequently used for a isolation of Nbs against large molecular antigens or in the case of lack free competitors [48,56]. The other is competitive elution, which is mostly applied to a selection of Nbs for small molecules, such as mycotoxins and chemical contaminants [53,55,57]. The construction of phage-displayed Nb library and the strategy of panning used to isolate high-affinity Nbs are shown in Figure 2.

Recently, a novel high-throughput screening strategy, “off-rate selection,” was proposed by Pírez-schirme et al. [58]. Nb specific to cyanotoxin microcystin-LR (995 Da) was obtained by promoting the isolation with the slowest k*off* for the immobilized hapten and exhibited higher sensitivity than Nbs selected by other two regular panning methods. This approach provides an alternative to improve the probability of positive anti-hapten Nb selection.

## 3. Performance of Nbs in the Field of Chemical Analysis

### 3.1. Affinity (Sensitivity) 

Affinity is the binding strength between an antigenic determinant and anantigen-binding site. Early studies mostly described low-affinity Nbs [59,60]. These results are not surprising, because the overall anti-hapten sdAbs response found in Camelide are observed to be modest. It may result from only a single variable domain of sdAb contributing to form the antigen binding site, which limits the contact between the antibody and antigen, whereas conventional antibodies appear to have a relatively two-dimensional shape [61]. Table 2 provides Nb assay sensitivities (represented by 50% inhibitory concentration, IC_50_) and, when possible, provides mAb or pAb-based assay sensitivities to the equivalent mycotoxin. The AFB_1_ and OTA detection were conducted by the method of indirect competitive enzyme-linked immunosorbent assay (icELISA) based on either Nb or conventional antibodies. As for 15-acetyl-DON determination, Nb-based competitive fluorescence polarization assay was developed and compared the sensitivity with mAb/pAb-based direct competitive ELISA (dcELISA). It was noted that Nbs have similar sensitivity in comparison with the conventional antibodies. In some cases, such as Nb against 2,2′,4,4′-tetrabromodiphenyl ether (BDE-47, molecular weight 485.79 Da) [62], the sensitivity of Nb-based icELISA (IC_50_ = 1.4 ng/mL) is even higher than that of mAb (IC_50_ = 1.75 ng/mL) [63]. In another study, Nb against tetrabromobisphenol-A (TBBPA, molecular weight 543.9 Da) [57], also exhibits higher sensitivity (IC_50_ = 0.4 ng/mL) by means of the icELISA method, compared with that of mAb (IC_50_ = 3.87 ng/mL) [64] and pAb (IC_50_ = 0.87 ng/mL) [65].

### 3.2. Selectivity

Nbs show high selectivity to macromolecular targets, especially as enzyme inhibitors due to their unique protruding CDR3, and show better selectivity toward closely related enzymes than conventional antibodies whose antigen-binding site is typically concave or flat and more likely to recognize the surface of the targets [72]. As for small haptens, it seems that no obvious difference between Nbs and conventional antibodies in terms of specificity, especially in recognizing antigens with structural analogues. The cross-reactivity of immunoassays is of usual occurrence, which is a common issue of both conventional antibodies and recombinant antibodies [60]. It was reported that the chance of selecting specific antibodies secreted from B cells mostly depends on the particular design of the immunized antigen, immunized dose, the ratio of the hapten and carrier protein and the spacer-arm lengths between the hapten and carrier protein [73,74,75,76]. However, it is feasible to produce specific Nbs towards a group of chemical analytes, such as Nb specific for OTA and its analogue, OTB, but no significant cross-reactivity for L-phenylalanine and coumarin, which have structural similarities with parts of OTA [77].

### 3.3. Stability

The stability of reagents is usually a key factor when developing immunoassay products. The reagents with a long shelf-life and high stability are always preferred. It has been reported that Nbs show greater resistance to heat than conventional antibodies and are thus more suitable to be applied as immunoreagents [44]. The main distinction between the Nb fragments and conventional antibody fragments is the reversibility of the thermal unfolding process, explaining its retained functionality after exposure to high temperatures [78]. Nbs have also been observed with high stability to organic solvents, which is beneficial for developing immunoassays to be used for the analysis of lipophilic analytes, such as mycotoxins and pesticides. Since the water-miscible solvents are commonly used for extraction of analytes prior to analysis, less dilution of the extracts is needed if the matrix effects for solvent extracts are negligible, enhancing the assay’s sensitivity. He et al. [55] estimated the solvent stability of Nbs against AFB_1_ with methanol, dimethylsulfoxide (DMSO), dimethylformamide (DMF), acetone, and acetonitrile. By comparison of the performance of mAb against AFB_1_, the results showed that Nbs displayed a higher stability to methanol, acetone, and acetonitrile than that of mAb.

### 3.4. Solubility

The single domain nature of Nbs leads them to facile single gene clone and easy to be expressed in soluble form in various expression systems, including bacteria, fungi, mammalian cell lines, plants, and insects [79]. The Nbs are naturally soluble because of the substitutions of hydrophobic by hydrophilic residues in the FR2. In addition, the absence of a linker peptide prone to aggregation or susceptible to proteolysis (e.g., as observed for scFv), promotes the solubility of Nb, even at concentrations above 10 mg/mL [80]. The majority of Nbs has been produced in *E. coli*, either periplasmically or cytoplasmically. Periplasmic Nbs production yields typically range from 1 to 10 mg/L while cytoplasmic Nbs production yields can reach 60–200 mg/L [79]. The *E. coli* strain containing Nb gene is feasible for reproduction and purification of soluble Nbs in shake flask cultures without immunizing animals, which is needed in mAbs and pAbs reproduction from host animals. The high solubility and high accumulation levels of Nbs make them ideal immunoreagents for the large-scale production in industry.

An overview of the Nbs presented within this review together with their targets, functional types, involved immunoassays and their performance are tabulated in Table 3.

## 4. Application of Nbs in the Area of Mycotoxin Detection

In spite of the unfavorable camelid immune response against small haptens, high-affinity Nbs to a diverse of targets have been generated and utilized to develop highly sensitive immunoassays for mycotoxin detection. Those methods, including Nb-based icELISA, one-step dcELISA based on Nb-alkaline phosphatase (AP) fusion protein, AI-Nb-based ELISA (AI-ELISA) and phage-Nb combined with immune-PCR. Some other exquisite designed immunoassays, such as Nb-based fluorescence polarization immunoassay and immunochromatographic assay, were also proved to be feasible, which widens the Nb application in mycotoxin detection in the area of food quality control.

### 4.1. Competitive Enzyme-Linked Immunosorbent Aassay 

**Figure 3:**
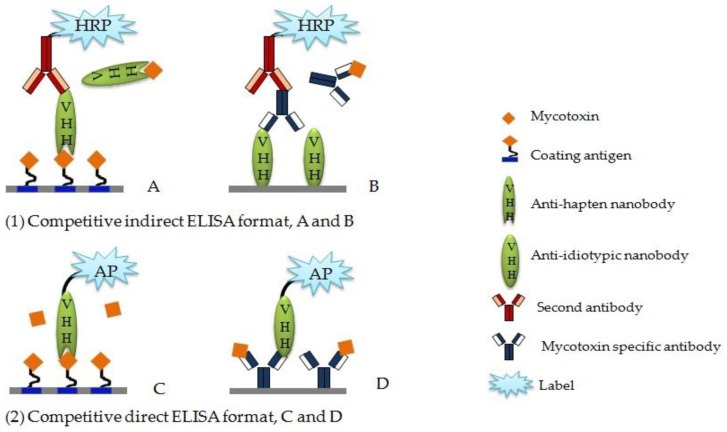
Schematic diagram of Nb-based competitive ELISA formats. The direct assays do not use second antibody and have one less step than the indirect assays. (**A**) Nb-based icELISA; (**B**) anti-idiotypic Nb-based icELISA, AI-ELISA; (**C**) Nb-based dcELISA; (**D**) anti-idiotypic Nb-based dcELISA, AI-dcELISA.

There are two normal types of competitive ELISA, indirect and direct competitive assay, applied in small chemical determination. Figure 3 displays the main forms of Nb-based competitive ELISA.

Anti-hapten Nbs can be used as affinity reagents in immune recognition and to construct a Nb-based icELISA (Figure 3A). Liu et al. [67] raised positive Nb against OTA and developed a sensitive Nb-based icELISA. The assay showed high resistance to cereal matrix interference and was applied to OTA-contaminated cereal samples determination. He et al. [55] developed an icELISA based on Nb for the analysis of AFB_1_, in which Nb also showed high stability under organic solvents and high temperature. The recovery of the assay was acceptable for the quantitative analysis of AFB_1_ in agro-product samples without dilution of extracted samples.

With the feasibility of genetic manipulation, Nb can be easily combined with alkaline phosphatase (Nb-AP) as fusion protein for direct colorimetric detection and construct the dcELISA (Figure 3C), which can avoid the chemical conjugation of a second enzyme and reduce the reaction step without altering sensitivity. Liu et al. [81] developed dcELISA based on a Nb-AP fusion protein for monitoring OTA contamination in cereal. The IC_50_ of the assay was 0.13 ng/mL, which increased 5-fold compared to Nb-based icELISA. Moreover, the detection limit and linear range were 0.04 ng/mL and 0.06-0.43 ng/mL, respectively. Shu et al. [49] developed a green and rapid one-step competitive enzyme immunoassay based on an AI-Nb and AP as fusion protein for FB_1_ detection in cereal (Figure 3D). The sensitivity of one-step dcELISA was 9-fold higher than two-step icELISA when the same isolated AI-Nb was involved in two types of ELISA.

### 4.2. Anti-Idiotypic Nb-based ELISA

Anti-idiotypic antibodies are novel antigens and generated according to primary antibodies, giving rise to humoral immune responses in syngeneic or xenogeneic systems [86]. The second antibody targets the antigenic determinants of the primary antibody, which can replace the conventional hapten-protein conjugates of small molecules, serving the same function in the competitive immunoassay. The Nbs against primary antibodies can be used as anti-idiotypic antibodies and work as non-toxic candidates for competing antigens in immunoassays [87] (Figure 3B).

Wang et al. [34] isolated AI-Nb from anti-aflatoxin mAb immunized alpaca library and then applied to immunoassay (AI-ELISA) toward aflatoxin detection as a coating antigen. The best immunoassay developed with the AI-Nb showed an IC_50_ value of 0.16 ng/mL toward AFB_1_ and cross-reactivity toward aflatoxin B_2_, G_1_, G_2_, and M_1_ of 90.4%, 54.4%, 37.7%, and 37.4%, respectively. The AI-ELISA was successfully applied to the aflatoxin analysis of peanuts, corn, and rice. The final assay has a good sensitivity and can be used to detect AFB_1_ in agro-products. The newly developed assay avoids the use of an aflatoxin standard in the synthesis of a hapten-protein conjugate as a coating antigen, which was replaced by an AI-Nb. 

Xu et al. [45,50] developed a simple and straightforward strategy to isolate AI-Nb as a CIT mimotope from a naive phage display Nb library. The obtained AI-Nb was successfully used as a competing antigen in establishing AI-ELISA and phage ELISA (AI-PELISA) for detection of CIT. The AI-ELISA exhibited dynamic linear detection of CIT from 5.0 to 300.0 ng/mL, with an IC_50_ value of 44.6 ng/mL, which was twice as good as that of icELISA (IC_50_ = 96.2 ng/mL) with CIT-OVA as a coating antigen. The AI-PELISA performed dynamic linear range from 2.5 ng/mL to 100.0 ng/mL with an IC_50_ value of 10.9 ng/mL, which was 9 times more sensitive than conventional CIT-OVA conjugates-based icELISA.

Qiu et al. [48] selected AI-Nb, N-28, which bind to anti-DON mAb from a naive phage display library and developed an AI-ELISA for sensitive detection of DON in cereals and feedstuffs. The IC_50_ value of the immunoassay developed was 8.77 ng/mL, which was 18-fold more sensitive than the conventional coating antigen (DON-BSA) based immunoassay. In addition, the interaction mechanism between isolated AI-Nb and anti-DON mAb was characterized and validated by molecular modeling and alanine-scanning mutagenesis. The results showed that hydrogen bond and hydrophobic interaction may contribute to their binding. Then, Qiu et al. [85] conducted site-saturation mutagenesis at five residues of N-28 and constructed a mutant phage display library. The AI-PELISA based on the best mutant N-28-T102Y was established and the IC_50_ value of the assay (IC_50_ = 24.49 ng/mL) was 3.2 times more sensitive than the wide-type N-28-based AI-PELISA (IC_50_ = 78.82 ng/mL). The linear range of the assay was 9.51–180.15 ng/mL. The established AI-PELISA was applied into DON detection in wheat, corn and feedstuff samples with good recovery values and the results of the real sample detection by AI-PELISA were in agreement with commercial ELISA kit.

In another work, Shu et al. [49] made use of an anti-FB_1_ mAb as the target for panning FB_1_ substitute from a naive alpaca Nb phage display library. The isolated AI-Nb was subjected to an AI-ELISA for the detection of FB_1_ contaminated in cereals and feedstuffs. The developed assay showed an IC_50_ value of 0.95 ng/mL, with a limit of detection of 0.15 ng/mL, linear range of 0.27–5.92 ng/mL, and low cross-reactivity toward FB_2_ (4.93%). The sensitivity of AI-ELISA was enhanced approximately 20-fold compared with that of the chemosynthetic FB_1_-BSA conjugates-based ELISA (IC_50_ = 21.14 ng/mL). The established AI-ELISA was validated to be suitable for monitoring the total fumonisin concentration under the current regulatory limits of fumonisins in most countries.

### 4.3. Phage Display Mediated Immuno-Polymerase Chain Reaction 

Phage display mediated immune-PCR (PD-IPCR) has been proved to be an ultrasensitive combined method using immunoassay and PCR for determination of toxins and markers. The phage-Nb and phage DNA can directly act as the detection antibody and PCR template, respectively.

**Figure 4:**
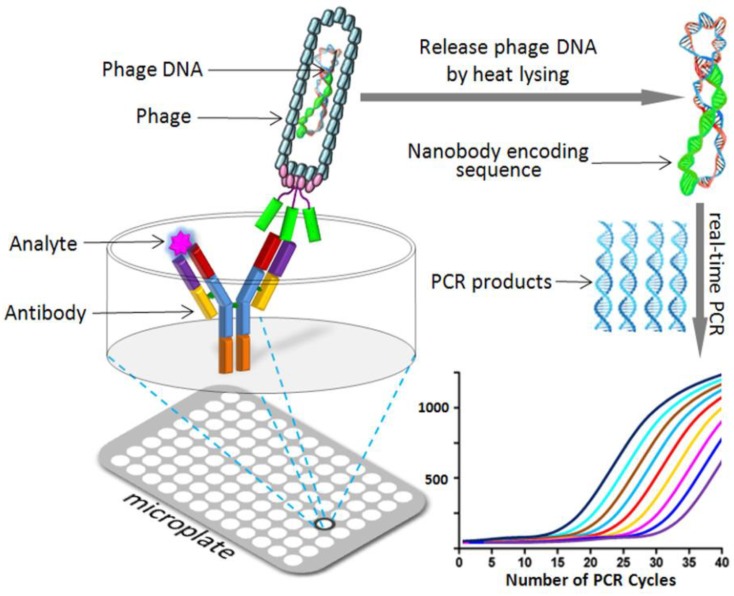
Schematic diagram of the real-time phage display mediated immune-PCR assay. (Reprinted with permission from reference [83], Copyright (2014), American Chemical Society.)

There are two forms of phage-borne Nb used to construct real-time IPCR (RT-IPCR) assay, one of which is antigen-specific Nb phage-based RT-IPCR (Nb-IPCR) and the other is AI-Nb phage-based RT-IPCR (AI-Nb-IPCR). The former phage-Nb is able to recognize immobilized antigen and compete with free analytes while the latter AI-Nb phage (phage-AI-Nb) against anti-hapten mAb can be used as surrogate for competitive antigen. Both of these two types of phage-displayed Nbs (phage-Nb and phage-AI-Nb) can be used as signal-amplification carrier with specific recombinant DNA to develop an environmentally friendly IPCR for chemical contaminant detection in agriculture and environment. Figure 4 shows the schematic diagram of the PD-IPCR assay format.

Liu et al. [82] generated OTA-specific Nb from an immunized alpaca Nb library and then applied phage-Nb to IPCR for ultrasensitive detection of OTA in cereal samples. The detection limit of the Nb-IPCR was 3.7 pg/L (LOD = 3.7 × 10^−6^ ng/mL), and with a wide linear range of 0.01–1000 pg/mL (LR: 10^−5^–1.0 ng/mL). The validation results indicated the reliability of Nb-IPCR in the detection of OTA in cereal samples. This method is the most sensitive assay reported to date for the detection of OTA and indicates that Nb-IPCR has great potential in the ultrasensitive detection of mycotoxins and other toxic small molecular compounds.

In another study, Ji et al. [47] developed a sensitive and non-toxin quantitative AI-Nb-IPCR for OTA based on phage-AI-Nb against anti-OTA mAb. The limit of detection of assay was 4.17 pg/mL, which exhibited a 9-fold improvement over AI-PELISA (LOD = 300 pg/mL). Moreover, a wide linear range (LR: 10–10,000 pg/mL) was available. The developed method was successfully validated with OTA contaminated agro-products.

Lei et al. [83] developed AI-Nb-IPCR assay for the accurately quantitative detection of major aflatoxins (AFB_1_, B_2_, G_1_ and G_2_) in agro-products based on phage-AI-Nb against anti-aflatoxins mAb. The limit of detection of the established assay was 0.02 ng/mL, which was 4-fold improvement over AI-PELISA (LOD = 0.08 ng/mL) for AFB_1_ detection. The developed method was successfully validated with the samples of corn, rice, peanut, and feedstuff. Then, Wang et al. [44] also developed AI-Nb-IPCR for ultrasensitive determination of ZEN in cereals. The limit of detection of the assay was 6.5 pg/mL, which was 12 times improved than that of exclusive AI-PELISA (LOD = 0.25 ng/mL), with a linear range of 0.01–100 ng/mL. The proposed method was successfully applied for the determination of ZEN in cereal samples.

### 4.4. Nb-Based Fluorescence Polarization Immunoassay

Doyle et al. [59] isolated Nb against 15-acetyl-DON from a hyper-immunized llama library, followed by soluble expression of monomeric and pentameric Nb for application in fluorescence polarization immunoassay. DON was conjugated with fluorescein and used as tracer. Serial dilutions of Nb were mixed with tracer to react in the dark and followed by fluorescence polarization analysis with excitation and emission wavelengths of 488 and 530 nm, respectively. A standard curve of competition between DON-fluorescein and free DON was prepared using a competitive fluorescence polarization assay. The assay inhibition with 15-acetyl-DON determined an IC_50_ value of 1.24 μΜ (419.5 ng/mL) and 0.50 μΜ (167.175 ng/mL) for monomer and pentamer Nb, respectively.

### 4.5. Nb-based Lateral-Flow Immunoassays

**Figure 5:**
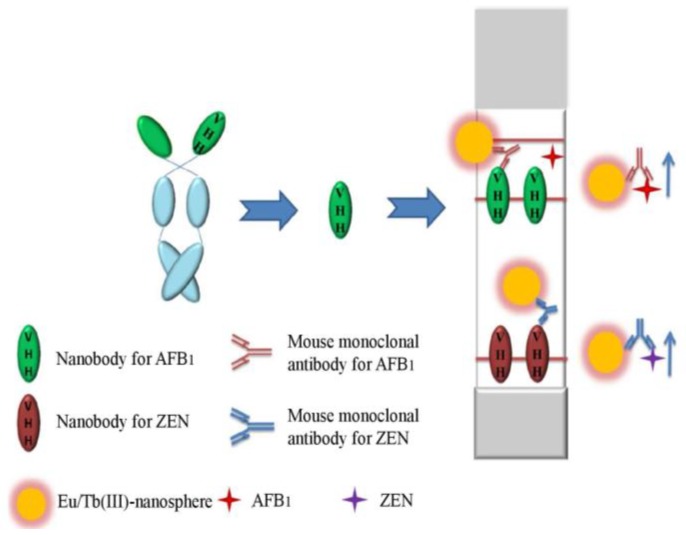
Schematic illustration of AI-Nb-based time-resolved fluorescence immunochromatographic assay for dual mycotoxins. (Reprinted with permission from reference [84], Copyright (2017), American Chemical Society.)

Antibody-based immunochromatographic assay is widely used in rapid detection of mycotoxins in agro-products. Tang et al. [84] developed time-resolved fluorescence immunochromatographic assay (TRFICA) using two AI-Nbs for rapid, quantitative, and simultaneous detection of AFB_1_ and ZEN in maize and its products. A novel lanthanides Eu/Tb(III) nanosphere with enhanced fluorescence was conjugated to two anti-hapten mAbs—one is against AFB_1_ and the other is against ZEN—and used as detectors. Two AI-Nbs, AFB_1_ surrogate and ZEN surrogate, serving as coating antigen and immobilized on two separating test lines as capture antigens. The pattern of competitive time-resolved strip methods (AI-Nb-TRFICA) was established and the standard carve was built. The IC_50_ value of AI-Nb-TRFICA was 0.46 and 0.86 ng/mL for AFB_1_ and ZEN, respectively. The simultaneous detection of dual mycotoxins by AI-Nb-TRFICA was established, which provided a quantitative relationship ranging from 0.13 to 4.54 ng/mL for AFB_1_ and 0.20 to 2.77 ng/mL for ZEN, with a detection limit of 0.05 and 0.07 ng/mL in the buffer solution, respectively. The assay showed good recoveries in samples and was applied to detect dual mycotoxins in maize samples with satisfied results. Figure 5 displays the schematic diagram of the AI-Nb-based TRFICA assay format.

This research is the first report about time-resolved strip method based on AI-Nb for dual mycotoxins detection, which provides evidence that the Nb reagent possesses a broad potential for a wide range of application in the field of food quality control in the future.

### 4.6. Nb-Based Immunoaffinity Method

Sample pretreatment and purification are usually needed previous to pollutants analysis. Antibody-based immunoaffinity method is a common tool used for samples clean-up. Nb has been proved to possess high degeneration resistance that would enable the purification reagent reuse and further replace conventional antibodies. Xiong et al. [88] applied anti-AFB_1_ Nb into magnetic-bead-based immunoaffinity extraction (M-IAE) method for purification of AFB_1_ from corn samples. Magnetic beads carrying poly (acrylic acid) brushes (MB@PAA) were fabricated as an “Nb container” for improving AFB_1_ adsorption capacity. The developed MB@PAA@Nb could be reused at least 10 times, without obvious loss of the capture efficiency for AFB_1_. This proposed MB@PAA@Nbs-based immunoaffinity extraction method is a highly promising, novel sample pre-treatment platform for AFB_1_ as well as other mycotoxins.

## 5. Challenge of Nb Application in Analytical Chemistry

Nbs have many favorable properties compared with conventional and other recombinant antibodies. The smaller number of CDRs does not seem to threaten the ability of Nbs to bind macromolecular antigens with high affinity, such as AI-Nb binding to mAb is easy to be obtained. However, the lack of a light chain possibly hampers their ability to bind small haptens that conventional antibodies typically recognize by the interplay of the VH and VL domains and six CDRs [89]. This is the case for the isolation of Nbs against small molecules, like mycotoxins.

Recently, only three anti-mycotoxin Nbs are generated. Failures in the generation of Nbs that recognize small haptens in solution are common [43]. It seems difficult to get out of dilemma even sophisticated screening routine operated [60]. The reason may be the camelid immune response against small molecules is predominated by conventional IgG1, with lower ratios of single domain subclasses of IgG2 and IgG3 [54]. Additional structural analysis of small molecule-Nb complexes would be helpful in understanding the interaction and leading a more rational design of immunizing and selecting haptens. However, there is very little information on the way in which Nbs bind small molecules. So far, only three crystal structures of hapten-Nb complexes have been revealed, corresponding to the azo dyes Reactive Red 1 (733 Da) [90], Reactive Red 6 (717 Da) [91], and the chemotherapy agent methotrexate (454 Da) [92]. Among these three complexes, all Nbs can provide cavities to accommodate haptens like the VH-VL crevice, even in the absence of a VL. The three CDRs usually contribute to form the groove except methotrexate, which is bound in a non-canonical way, being deeply buried in a “tunnel” roofed by CDR1 and a loop of FR3 that the authors identified as “CDR4”. As a result, more efforts are needed to generate Nbs against small haptens and to further determine the structure characteristic and molecular recognition mechanism. Owing to their small size and ease of expression, Nbs are usually easy to crystallize or operate nuclear magnetic resonance (NMR) determination for structure study. It suggests that more Nb structures may be elucidated soon. Those emerging structures will help to identify the possible mechanisms and patterns of the interaction between Nbs and their targets, especially as to Nbs recognizing small haptens. Understanding the structure of the combining pocket will help to adjust Nbs characteristics through in vitro antibody maturation techniques, and in addition, to guide artificial antigens design rationally.

Additionally, Nb-based immunodetection formats are still limited and need to be further explored for mycotoxin detection, such as biosensors and portable devices [93,94]. It has been reported that the anti-hapten Nbs can be employed as recognition element and incorporated into two biosensor formats. The Nb can be coated on the surface of an electrode for electrochemical impedance detection [62], and coated onto a PDMS membrane for a lab-on-a-chip sensor [95]. These studies demonstrate the potential of developing novel and reliable assays for mycotoxin detection by Nb technology.

## 6. Conclusions

Ever since their discovery, Nbs have emerged as powerful antigen binders and have become a very promising tool for the monitoring of food quality. In this review, we summarized the recent advancements of Nb-based immunoassays for mycotoxin detection, including indirect competitive ELISA, direct competitive ELISA, phage-displayed Nb-mediated immuno-PCR, fluorescence polarization immunoassay, immunochromatographic assay, and immunoaffinity methods. Lots of studies have demonstrated Nb technology to be a powerful and potential technique for advanced applications in mycotoxin detection. As already mentioned, Nbs have striking properties such as high solubility, ease of genetic manipulation, and high thermal and conformational stability. However, it is still very challenging to select desirable Nbs binding to the small molecule epitopes from the phage display Nb repertoire, hampering the more extensive applications of Nbs in mycotoxin detection. Efforts should be made as follows to cope with the difficulties: (1) improve the success rate of Nb generation. It is necessary to ensure the immunogenicity of the designed hapten-protein conjugate, the capacity and diversity of the constructed Nb repertoire, as well as the optimization of the screening strategy; (2) the elucidation of the structure of Nbs and their complex with small molecules is a promising way to modify the properties of Nbs and to provide clues to the rational design of immunogen for the anti-hapten Nbs generation; and (3) innovative immunodetection formats should be further exploited to improve the performance of Nb-based assay and to expand the applicability of Nbs for the mycotoxin detection.

## Figures and Tables

**Table 1:** The major fungal producers, hosts, toxic effects of mycotoxins, and their maximum permitted levels in food according to European legislation.

Mycotoxins	Major Producers	Major Host	Toxin Effects	Maximum Level
Aflatoxins (AFB_1_, AFB_2_, AFG_1_, AFG_2_)	*Aspergillus flavus*, *A. parasiticus*	maize, nuts, cottonseed, peanuts	hepatotoxicity, cancer, immunosuppression	8.0 μg/kg (AFB_1_); 15 μg/kg (total aflatoxins) (Commission Regulation, EU No.165/2010) [12]
Fumonisins (FB_1_, FB_2_)	*Fusarium verticillioides*, *F. proliferatum*	maize	hepatotoxicity, cancer, pulmonary edema, leukoencephalomalacia	4000 μg/kg in maize; 1000 μg/kg in maize-based foods (EC No.126/2007) [13]
Deoxynivalenol (DON)	*F.graminearum*, *F. culmorum*	maize, wheat, barley	gastrointestinal toxicity, inflammation of central nervous system	1250 μg/kg in cereals (EC No.1126/2007)
Zearalenone (ZEN)	*F. culmorum*, *F.graminearum*	maize, barley, wheat, rice	infertility, abortion	350 μg/kg in maize; 100 μg/kg in other cereals (EC No.1126/2007)
OchratoxinA (OTA)	*Penicillium verrucosum*, *A.ochraceus*	cereal-derived products, spices, wine	nephrotoxic, carcinogenic, teratogenic, immunotoxic effects	5.0 μg/kg in maize (EC No.1881/2006)
Citrinin (CIT)	*P.citrinum*, *P. verruscosum*, *P.expansum, A.terreus*, *Monascus ruber*	corn, wheat, barley, rice	nephrotoxic, hepatotoxic, immunotoxic, carcinogenic effects	2000 μg/kg (EU No.212/2014) [14]

**Table 2:** Nbs to mycotoxins to date and comparison of sensitivities to monoclonal antibodies and polyclonal antibodies.

Mycotoxin	Antibody	Assay Format	Sensitivity, IC_50_
AFB_1_	Nb	icELISA [55]	0.754 ng/mL
	mAb	icELISA [17]	0.0002 ng/mL
	pAb	icELISA [66]	2.0 ng/mL
OTA	Nb	icELISA [67]	0.64 ng/mL
	mAb	icELISA [68]	0.058 ng/mL
	pAb	icELISA [69]	5.0 ng/mL
15-acetyl-DON	Nb	fluorescence polarization [59]	419 ng/mL
	mAb	dcELISA [70]	1000 ng/mL
	pAb	dcELISA [71]	1.9 ng/mL

**Table 3:** Some applications and the performance of Nbs used for mycotoxin detection.

Targets (MW).	NB TYPE	Assay Formats, (Ref.)	Sensitivity, IC_50_; Linear Range, IC_20_–IC_80_; LOD, IC_10_	Specificity (CR)	Thermal Stability	Solvent/Matrix Stability
OTA (403.813)	Anti-hapten Nb	icELISA [67]	IC_50_: 0.64 ng/mL; LR: 0.27–1.47 ng/mL; LOD: 0.16 ng/mL	not tested	95 °C for 5min, retained45% of binding activity; 90 °C for 75 min, retained25%of binding activity	dilution factor ×2.5 eliminate the matrix interference
	Nb-AP	dcELISA [81]	IC_50_: 0.13 ng/mL; LR: 0.06–0.43 ng/mL; LOD: 0.04 ng/mL,	CR: 0.1% with OTB, ZEN, DON, and AFB_1_	not tested	Susceptive to methanol, ionic strength and low pH (≤6.0)
	phage-Nb	Nb-IPCR [82]	LR: 10^−5^–1.0 ng/mL; LOD: 3.7 × 10^−6^ ng/mL;	CR: 3.5% with OTB; 0.1% with FB_1_, ZEN, DON, and AFB_1_	not tested	not tested
	phage-AI-Nb	AI-Nb-IPCR [47]	LR: 0.01–10 ng/mL; LOD: 0.004 ng/mL	CR: 0.1% with FB_1_, ZEN, DON, and AFB_1_	not tested	susceptive to methanol over 10%
AFB_1_ (312.27)	Anti-hapten Nb	icELISA [55]	IC_50_: 0.754 ng/mL; LR: 0.117–5.676 ng/mL; LOD: 0.05 ng/mL	CR: 10% with AFB_2_, AFG_1_, and AFG_2_	85 °C for 60 min, retained40% of binding activity	stable in 40% methanol and 40% acetone
	phage-AI-Nb	AI-Nb-IPCR [83]	LOD: 0.02 ng/mL	CR: 50% with AFB_2_ and AFG_1_; 13.5% with AFG_2_	not tested	susceptive to methanol over 10%
	AI-Nb	AI-ELISA [34]	IC_50_: 0.16 ng/mL; LR: 0.09–0.82 ng/mL; LOD: 0.06 ng/mL	CR: 50% with AFB_2_ and AFG_1_; 30% with AFG_2_ and AFM_1_	80 °C for 60 min, retained 20% of bindingactivity	stable in methanol below 40%
	AI-Nb	TRFICA [84]	IC_50_: 0.46 ng/mL; LR: 0.13–4.54 ng/mL; LOD: 0.05 ng/mL	CR: 50% with AFB_2_ and AFG_1_; 31.1% with AFG_2_; 19.4% with AFM_1_	not tested	not tested
ZEN (318.37)	phage-AI-Nb	AI-Nb-IPCR [46]	LR: 0.01–10 ng/mL; LOD: 0.0065 ng/mL	CR: 0.1% with AFB_1_, DON, and OTA	not tested	susceptive to methanol over 5%
	AI-Nb	TRFICA [84]	IC_50_: 0.86 ng/mL; LR: 0.20–2.77 ng/mL; LOD: 0.07 ng/mL;	CR: 78.1% with β-zearalenol	not tested	not tested
15-acetyl-DON (338.35)	Anti-hapten Nb	fluorescence polarization [59]	IC_50_: 419.5 ng/mL (monomer) 167.175 ng/mL (pentamer)	CR: 0.1% with neosolaniol, diacetoxyscirpenol, and T-2 toxin	not tested	not tested
DON (296.32)	AI-Nb	AI-ELISA [48]	IC_50_: 8.77 ng/mL; LR: 2.18–62.25 ng/mL; LOD: 1.16 ng/mL	CR: 0.1% with FB_1_, ZEN, AFB_1_, and OTA	95 °C for 5 min, retained60% of binding activity	not tested
	phage-AI-Nb	AI-PELISA [85]	IC_50_: 24.49 ng/mL; LR: 9.51–180.15 ng/mL	CR: 0.1%with FB_1_, ZEN, AFB_1_, and OTA	95 °C for 5 min, retained55% of binding activity	stable in ionic strength below 50 mM and pH between 5.0 and 8.0
FB_1_ (721.84)	AI-Nb	AI-ELISA [49]	IC_50_: 0.95 ng/mL; LR: 0.27–5.92 ng/mL; LOD: 0.15 ng/mL	CR: 4.93% with FB_2_	not tested	stable in ionic strength below 50 mM and pH between 5.0 and 8.0
CIT (250.25)	AI-Nb	AI-ELISA [50]	IC_50_: 44.6 ng/mL; LR: 5.0–300.0 ng/mL;	CR: 0.1% with AFB_1_, ZEN, DON, and OTA	not tested	susceptive to methanol over 20%
	phage-AI-Nb	AI-PELISA [45]	IC_50_: 10.9 ng/mL; LR: 2.5–100.0 ng/mL	CR: 0.1% with AFB_1_, ZEN, DON, and OTA	not tested	stable in methanol below 25%, ionic strength below 55 mM and pH between 5.4 and 9.0

Abbreviations used: (1) MW, molecular weight; (2) IC_50_, IC_20_–IC_80_, IC_10_, concentrations resulting in 50, 20-80 or 10% decrease in maximum signal; (3) LR, linear range; (4) LOD, limit of detection; (5) CR, cross-reactivity; (6) ELISA, enzyme linked immunosorbent assay; (7) ic-ELISA, indirect competitive ELISA; (8) dc-ELISA, direct competitive ELISA; (9) Nb-IPCR, Nb-based phage displayed immune PCR; (10) AI-Nb-IPCR, anti-idiotypic Nb based phage displayed immune PCR; (11) AI-ELISA, anti-idiotypic Nb based ELISA; (12) AI-PELISA, anti-idiotypic Nb-based phage ELISA; (13) TRFICA, time-resolved fluorescence immunochromatographic assay; (14) Nb-AP, Nb-alkaline phosphatase fusion protein; (15) phage-Nb, phage-displayed Nb; (16) phage-AI-Nb, phage-displayed anti-idiotypic Nb. * units converted to ng/mL from initial publication.
